# Reproductive isolation is mediated by pollen incompatibility in sympatric populations of two *Arnebia* species

**DOI:** 10.1002/ece3.1849

**Published:** 2015-11-26

**Authors:** Lin‐Lin Wang, Chan Zhang, Bin Tian, Xu‐Dong Sun, Wen Guo, Ting‐Feng Zhang, Yong‐Ping Yang, Yuan‐Wen Duan

**Affiliations:** ^1^ Key Laboratory for Plant Diversity and Biogeography of East AsiaKunming Institute of BotanyChinese Academy of SciencesKunming650201China; ^2^ Plant Germplasm and Genomics Centerthe Germplasm Bank of Wild SpeciesKunming Institute of BotanyChinese Academy of SciencesKunming650201China; ^3^ Institute of Tibetan Plateau Research at KunmingKunming Institute of BotanyChinese Academy of SciencesKunming650201China; ^4^ College of Life SciencesHenan Normal UniversityXinxiang453007HenanChina; ^5^ Key Laboratory of Biodiversity Conservation in Southwest China of State Forestry AdministrationSouthwest Forestry UniversityKunming650224YunnanChina; ^6^ College of Agriculture and BiotechnologyHexi UniversityZhangye734000GansuChina

**Keywords:** Floral traits, natural selection, pollen export, pollinator preference, seed set

## Abstract

To explore uncertain aspects of the processes that maintain species boundaries, we evaluated contributions of pre‐ and postpollination reproductive isolation mechanisms in sympatric populations of *Arnebia guttata* and *A. szechenyi*. For this, we investigated their phylogenetic relationships, traits, microenvironments, pollinator visits, action of natural selection on floral traits, and the outcome of hand pollination between the two species. Phylogenetic analysis indicates that *A. szechenyi* is a derived species that could be closely related to *A. guttata*, and both could be diploid species. *Arnebia guttata* flowers have larger parts than *A. szechenyi* flowers, but smaller nectar guides. Soil supporting *A. szechenyi* had higher water contents than soil supporting neighboring populations of *A. guttata* (in accordance with their geographical distributions). The pollinators shared by the two species preferred *A. szechenyi* flowers, but interspecific visitations were frequent. We found evidence of conflicting selection pressures on floral tube length, flower diameter and nectar guide size mediated via male fitness, and on flower diameter and floral tube diameter via female fitness. Hand‐pollination experiments indicate complete pollen incompatibility between the two species. Our results suggest that postpollination prezygotic mechanisms are largely responsible for reproductive isolation of sympatric populations of the two *Arnebia* species.

## Introduction

Speciation, the division of populations into evolutionarily independent lineages, is a fundamental evolutionary process. There are caveats regarding the strict validity of the species concept (De Queiroz 2007). However, if the general concept (biological species) is accepted, the key process in the formation and maintenance of species is the evolution of reproductive isolation, that is, mechanisms that severely restrict gene flow between formerly interbreeding populations (Mayr 1942). Elucidation of these mechanisms requires quantitative analysis of the factors contributing to reproductive isolation between pairs of species with very close phylogenetic relationships, often known as sister species (Moyle et al. 2004).

Generally, reproductive isolation of sister species of plants involves various pre‐ and postzygotic barriers (Coyne and Orr 2004; Butlin et al. 2012). However, postzygotic isolation is costly due to associated gamete wastages. Thus, prezygotic isolation mechanisms should be more important than postzygotic isolation mechanisms for sympatric species (Baack et al. 2015), although the latter dominate in some cases (Costa et al. 2007). Prezygotic reproductive isolation can be further classified as pre‐ and postpollination. For sympatric plants, prepollination isolation may involve differences in flowering phenology (Pascarella 2007), pollinator fidelity (Ramsey et al. 2003), and/or variations in mating systems (Brys et al. 2014), while postpollination isolation may involve gamete incompatibility and/or pollen tube competition (Coyne and Orr 2004).

**Figure 1:**
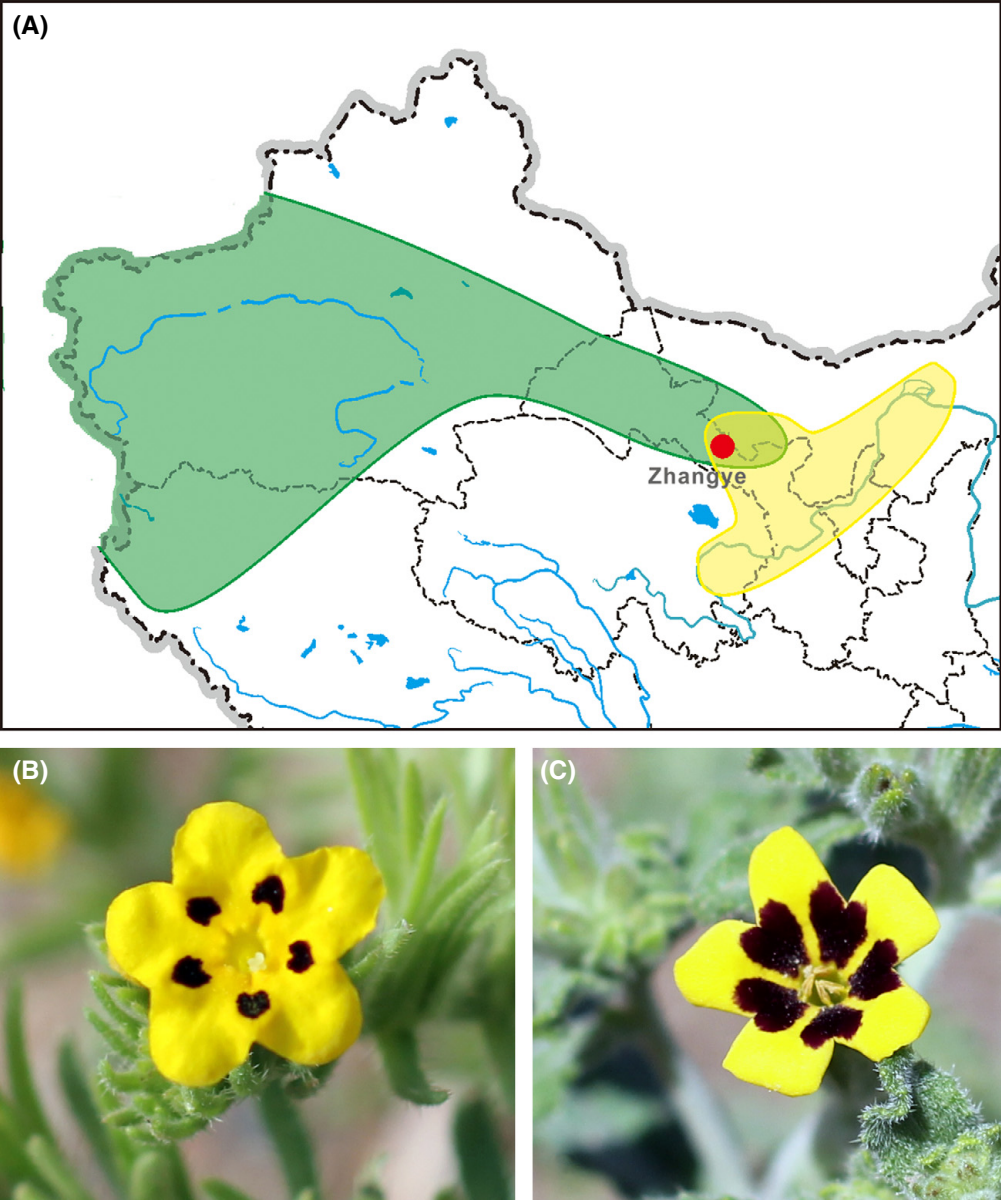
Geographical distributions (A) of *A. guttata* (green) and *A. szechenyi* (yellow) in China based on herbarium and field investigations, and photographs of flowers of *A. guttata* (B) and *A. szechenyi* (C). The red dot indicates the study site in the field.

In the study presented here, we evaluated contributions of pre‐ and postpollination mechanisms to reproductive isolation in sympatric populations of two *Arnebia* species (*A. guttata* and *A. szechenyi*, Boraginaceae) (Fig. 1A). We first examined the phylogenetic relationship between the two species to determine whether or not they are sister species, then examined their ploidy levels to identify whether or not there is a ploidy‐based isolating barrier (Husband and Sabara 2004; Thompson and Merg 2008). We also investigated differences in their floral traits, pollinator visits, pollinator‐mediated selection, and microhabitats in the field. Our main objectives were to evaluate pollinator discrimination and selection of the two species and to determine the importance of pre‐ and postzygotic barriers in their reproductive isolation.

## Materials and Methods

### Plant species and study sites

*Arnebia* is a small genus with approximately 25 species, including six species in China (Zhu et al. 2005). Characteristics of the genus include distyly and heteromorphic incompatibility, and thus, all of its members require a pollinator for seed production (Feng and Tan 2006; Zhang et al. 2014). *Arnebia guttata* inhabits the Gobi Desert and rocky slopes in northern China and central Asia, while *A. szechenyi* is one of two species that are endemic to China (the other being *A. tschimganica*) and usually inhabits sunny mountain slopes along the Yellow River (Zhu et al. 2005). We found a significant overlap in these two species’ ranges from northwest Gansu to southern Inner Mongolia during field expeditions (Fig. 1A). Thus, as they met key criteria for quantifying reproductive isolation processes, we explored mechanisms of pre‐ and postzygotic reproductive isolation in sympatric populations of *A. guttata* and *A. szechenyi*, in field experiments and phylogenetic analyses, as described below.

Our field experiments were conducted near the city of Zhangye (39°4′ N, 100°25′ E, 1430 m), northwest Gansu province, from May to June in 2013, focusing on populations of *A. guttata* and *A. szechenyi* located approximately 200 m from one another, designated AG‐Z and AS‐Z, respectively (Table S1). The freshly opened flowers of *A. guttata* and *A. szechenyi* are similar morphologically, and each exhibits five black patches on a yellow corolla (Fig. 1B and C), which could potentially act as nectar guides. These patches disappear on the second day after the flowers open for unknown reasons, but there is no difference in appearance between flowers with and without nectar guides in images captured using a camera equipped with a UV lens (not shown) (our unpublished data).

### Phylogenetic relationship

Twelve individuals of *A. szechenyi* and *A. guttata* were sampled from the sympatric AS‐Z and AG‐Z populations, respectively, and further sets of 12 from sympatric populations designated AS‐Y and AG‐Y, respectively, near Yabulai town, Inner Mongolia (Table S1). We also collected specimens of the congeneric species *A. fimbriata* for phylogenetic analyses. Total genomic DNA was extracted from leaves of each individual, and the primers ITS1 (internal transcribed spacer 1) (5′‐TCCGTAGGTGAACCTGCGG‐3′) and ITS4 (5′‐TCCTCCGCTTATTGATATGC‐3′) were used to amplify ITS sequences (White et al. 1990). The PCR products were purified and then sequenced in an ABI 3730 automated sequencer. The obtained sequences were aligned using MUSCLE (Edgar 2004), as implemented in MEGA 5.0 (Tamura et al. 2011). Corresponding sequences of other species of *Arnebia* (*A. euchromae*,* A. decumbens*,* A. linearifolia*) and *Onosma paniculatum* were also downloaded from GenBank (Table S1) for the analyses. We determined haplotype phases of ITS using PHASE version 2.1.1 (Stephens et al. 2001) and examined phylogenetic relationships of the different ITS sequences through maximum parsimony (MP) analyses using PAUP* version 4.0 (Swofford 2002). We calculated bootstrap percentages (BP) using 1000 replicates (Felsenstein 1985) and employed MrModeltest 2.0 to choose the most appropriate model for each dataset for maximum likelihood (ML) analyses (Nylander 2004). The ML analyses were performed in PHYML 3.0 (Guindon et al. 2010) with 1000 bootstrap replicates under the GTRIG model (Guindon and Gascuel 2003).

### Divergence in species’ traits and microhabitats

We investigated differences in floral traits, ploidy levels, microhabitats, and root micromorphology between the two *Arnebia* species to quantify their divergence. To quantify the differences in floral traits, we randomly selected newly opened flowers of different plants and measured the length of the corolla tube, diameter of the corolla, diameter of the corolla tube, size (width) of the nectar guide, and outer diameter of the nectar guide circle using a digital caliper. Two‐way ANOVA was applied to compare flower traits, with floral type (long‐ or short‐style) and species (*A. guttata* and *A. szechenyi*) as fixed factors.

To determine the ploidy levels in *A. guttata* and *A. szechenyi*, we randomly collected mature seeds from 10 plants of each species. All seeds were soaked in distilled water at room temperature (approximately 25°C) until germination. *Arnebia guttata* is reportedly a diploid species (Fang and Zhang 1992), thus we used this species as a reference to examine the ploidy level in sympatric *A. szechenyi* using flow cytometry, as follows. When roots of the germinated seeds were approximately 5 cm long, the hypocotyls were separated and crushed in a Petri dish containing 2 mL prechilled lysis buffer (Dolezel et al. 2007). After filtration, centrifugation, re‐suspension and storage in the dark at 4°C for 30 min, the resulting cell suspensions were analyzed using a FACS‐Vantage flow cytometer following the manufacturer's recommendations (Partec, German). The ploidy level of *A. szechenyi* was estimated by comparing the mean fluorescence intensity of the nuclei of the sample material with that in the reference standard. In addition, root tips of the seedlings were removed and fixed in a 2.5% glutaraldehyde solution to examine their micromorphology.

To assess root micromorphology, fixed roots were washed three times with 0.1 mol/L phosphate buffer solution within 30 min, and then serially dehydrated for 6 ‐ 8 min in a series of 30, 50, 70, 85, 95, and 100% ethanol. The specimens were infiltrated in low‐viscosity resin, then 1 *μ*m thick sections were taken with a Leica EM UC6 microtome and stained with 0.5% toluidine blue. The sections were observed under bright‐field optics using a Leica DM 1000 microscope (Sun et al. 2013).

The microhabitats of the two *Arnebia* species were compared in terms of the water content of the soils, by taking samples from 10 cm below ground level at microsites of each of 10 plants of each species. Each sample was placed in a preweighed aluminum container with a cap and heated at 80°C in an oven to constant weight. The weights of the capped container before addition of the sample and both before and after heating (measured to within ±0.001 g using an electronic balance) were then used to calculate the dry mass and water content of each sample. Finally, the water content of the soil supporting the two species was compared using independent‐*T* tests.

### Pollinator visits and hand‐pollination experiments

To compare pollination characteristics of the two *Arnebia* species, we observed visitors to identify each species’ pollinators and determine whether any of them visited both species, as follows. On sunny days without strong wind we randomly selected several plants of each species and recorded the numbers of flowers with nectar guides as our preliminary observations suggested that pollinators almost always visit flowers with nectar guides. Then, we observed the visitors to the flowers and recorded whether or not they touched the anthers and stigma of the flowers to determine if they were potential pollinators. In total, we observed 22 plants of *A. guttata* and 17 plants of *A. szechenyi* for 42 and 41 h, respectively. We then applied two‐way ANOVA to analyze differences in pollinator visitation rates, with plant species and pollinator as fixed factors.

All of the identified pollinators moved too quickly to be traced. Thus, in order to examine pollinator fidelity, we performed reciprocal transplant experiments to evaluate the potential for interspecific visitations by the shared pollinators. On each day before observations, we reciprocally transplanted 4–5 plants of one species into the population of the other species and selected the same number of plant individuals of the other species adjacent to the transplanted plants. All the transplanted plants were placed in tubes filled with water to keep them fresh during the observation periods. Then, we observed these plants and recorded intra‐ and interspecific visitations. In total, we observed 85 pollinators visiting 21 plants of each *Arnebia* species during 23 h of observation over 5 days.

To evaluate the level of pollen compatibility, or incompatibility between *A. guttata* and *A. szechenyi*, we performed hand‐pollination experiments in the field. For each species, we selected 60 long‐styled and 60 short‐styled flower buds from different plants of each species and separated each set into three groups. All of the flowers were emasculated and bagged prior to opening. After the flowers opened, the three groups of flowers were subjected to hand pollination by pollen from: (1) flowers of the same species but different morphology (style length); (2) flowers of the other species and different morphology; and (3) flowers of the other species with the same morphology. We excluded pollination with pollen from flowers of the same species and same morphology because of the known distyly and heteromorphic incompatibility of the genus (Feng and Tan 2006; Zhang et al. 2014). Eight hours after hand pollination, the stigmas of five flowers from each group were collected and fixed in FAA solution (formalin:acetic acid:100% ethanol at a ratio of 5:5:90 by volume) for observations of pollen tube growth, and the remaining flowers were left to set seeds. In the laboratory, the stigmas were softened in 8 mol/L NaOH for 8 h, then stained by incubation in aniline blue solution (1%) after immersion in distilled water for 3 h (Dafni et al. 2005). Each stigma was then squashed on a slide, and pollen tube growth was observed with a fluorescence microscope.

### Natural selection pressure acting on floral traits

To determine whether or not selection pressures acting on floral traits of the two *Arnebia* species differ, we evaluated the pressures from male fitness and female fitness values based on pollen exports and numbers of ovules that developed, respectively. Flower buds from 50 long‐style plants and 50 short‐styled plants of each species were selected from each species, tagged, and subjected to open pollination. Using a digital caliper, we measured the length of the corolla tube, diameter of the corolla, diameter of the corolla tube, width of the nectar guide, and diameter of the outer nectar guide circle of each selected flower after it opened. When the flowers began to wilt, we collected all of the anthers of each flower and fixed them in FAA to determine the number of pollen grains retained in the flowers. Ten buds of long‐ and short‐styled flowers from different plants of each species were also fixed in FAA to determine a baseline for the total number of pollen grains per flower. In the laboratory, we squashed all of the anthers from each flower and suspended all of the pollen grains they contained in 5 mL of water with a drop of detergent solution to full suspension. The total number of pollen grains retained in each flower was determined by counting the number of pollen grains in 20 drops (2 *μ*L) of pollen solution under a light microscope (Dafni et al. 2005). Using these data we calculated the number of pollen grains exported to estimate male fitness. Two weeks after the tagged flowers wilted, the number of developed ovules in each flower was determined.

To estimate the natural selection pressure acting on floral traits via male fitness, we employed multiple regression analyses with relative male fitness as the response variable and the standardized trait values as explanatory variables (Lande and Arnold 1983). Both the pollen export and seed set values were transformed into corresponding relative fitness (individual fitness divided by mean fitness) values, and floral traits were standardized (by mean centering and scaling to unit variance). However, the number of ovules was constant (four) across all of the flowers in both species, so we employed logistic regression analysis to estimate the natural selection pressure acting on floral traits via female fitness (Janzen and Stern 1998). Initially we included quadratic terms (*γ*) to quantify nonlinear selection pressure, but none of the quadratic gradients was statistically significant. Therefore we only report linear gradients here.

## Results

### Phylogenetic relationship

**Figure 2:**
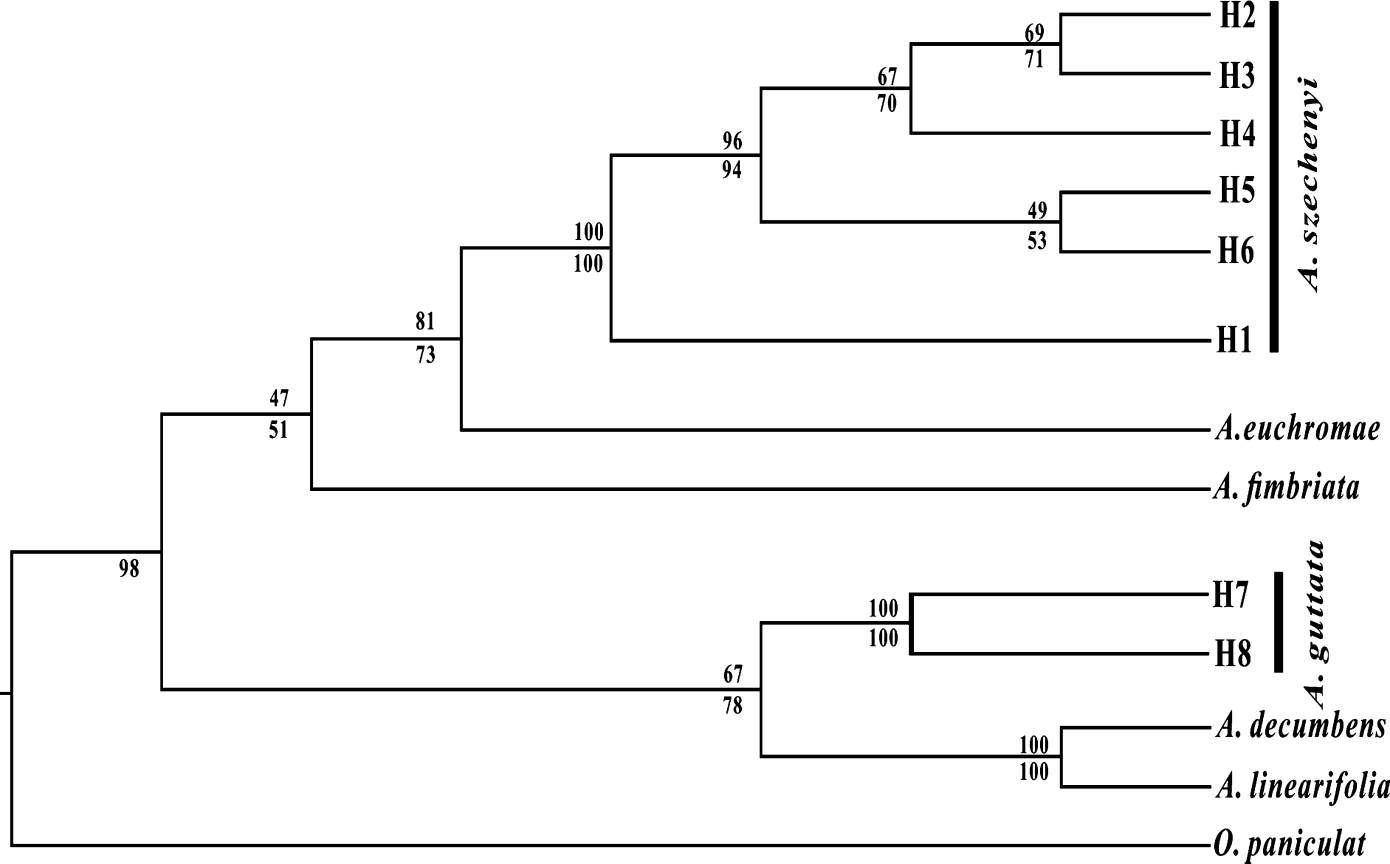
Phylogenetic trees reconstructed using the maximum likelihood method based on the nrDNA ITS matrix including extra sequences from additional individuals. Bootstrap support values from the ML analyses are shown below the branches and values from MP analyses above the branches.

The aligned ITS sequences were 650 bp long. Two different ITS sequences were detected in *A. guttata*, and six in *A. szechenyi* (Table S1). The topologies produced from the ML and MP analyses were largely consistent. Thus, only the ML tree is presented here. The nuclear DNA analysis indicated two major clades: one with low support formed by *A. fimbriata*,* A. euchroma,* and all *A. szechenyi* haplotypes; and another with moderate support formed by *A. linearifolia*,* A. decumbens,* and all *A. guttata* haplotypes (Fig. 2).

### Divergence in species traits and microhabitats

Of the five measured floral traits, the nectar guide was significantly smaller but the others were significantly larger in *A. guttata* than in *A. szechenyi* (Table 1). Generally, the five measured floral traits differed significantly between the two species, but three were also significantly affected by floral type, and two were significantly affected by species×floral type interaction (Table 2). In addition, our results suggest that the chromosome number of *A. szchenyi* is 2n = 14, but the chromosomes of this species were too small to discern clearly in histochemical preparations (not shown). In the flow cytometric analysis, the *A. szechenyi* samples yielded flow histograms with a single peak (112.35 ± 7.00%), similar to *A. guttata* samples (101.49 ± 7.50%), further indicating that *A. szechenyi* is a diploid species.

**Table 1:** Floral traits (Mean ± SE) of the two *Arnebia* species. Numbers in brackets are the sample sizes

Floral traits (mm)	*A. guttata*		*A. szechenyi*	
	Long‐styled (66)	Short‐styled (88)	Long‐styled (100)	Short‐styled (97)
Floral tube length	15.69 ± 0.16	16.41 ± 0.18	11.79 ± 0.10	12.58 ± 0.09
Flower diameter	13.44 ± 0.14	13.35 ± 0.15	10.61 ± 0.12	10.82 ± 0.08
Floral tube diameter	1.56 ± 0.03	2.50 ± 0.12	1.18 ± 0.02	1.61 ± 0.03
Nectar guide size	1.65 ± 0.04	1.56 ± 0.03	2.74 ± 0.04	2.54 ± 0.03
Nectar circle diameter	6.71 ± 0.08	6.39 ± 0.08	5.57 ± 0.07	5.61 ± 0.05

**Table 2:** Results of two‐way ANOVA of the differences between the floral traits, with the species and floral type (long‐ and short‐styled) as fixed factors

	Species	Floral type	Interaction
Floral tube length	789.71^a^	30.37^a^	1.41
Flower diameter	423.15^a^	0.50	2.14
Floral tube diameter	310.08^a^	347.33^a^	12.83^a^
Nectar guide size	844.91^a^	13.41^a^	0.35
Nectar circle diameter	171.42^a^	3.25	6.90^a^

a *P* < 0.01.

The cross sections of the *A. guttata* and *A. szechenyi* roots revealed a clear bicollateral arrangement of vascular tissue, with the xylem between two areas of phloem (Fig. S2). Vessel cells are larger in *A. guttata* (Fig. S2A, C) than in *A. szechenyi* (Fig. S2B, D) (1.27 ± 0.01 µm vs. 0.44 ± 0.02 µm in width, *t* = 30.66, df = 18, P < 0.001). Correspondingly, the water content of the soil was significantly lower around *A. guttata* plants than around *A. szechenyi* plants (1.12 ± 0.08 and 2.19 ± 0.14%, respectively; *t* = 6.6, df = 18, P < 0.001).

### Pollinator visits and hand‐pollination results

**Figure 3:**
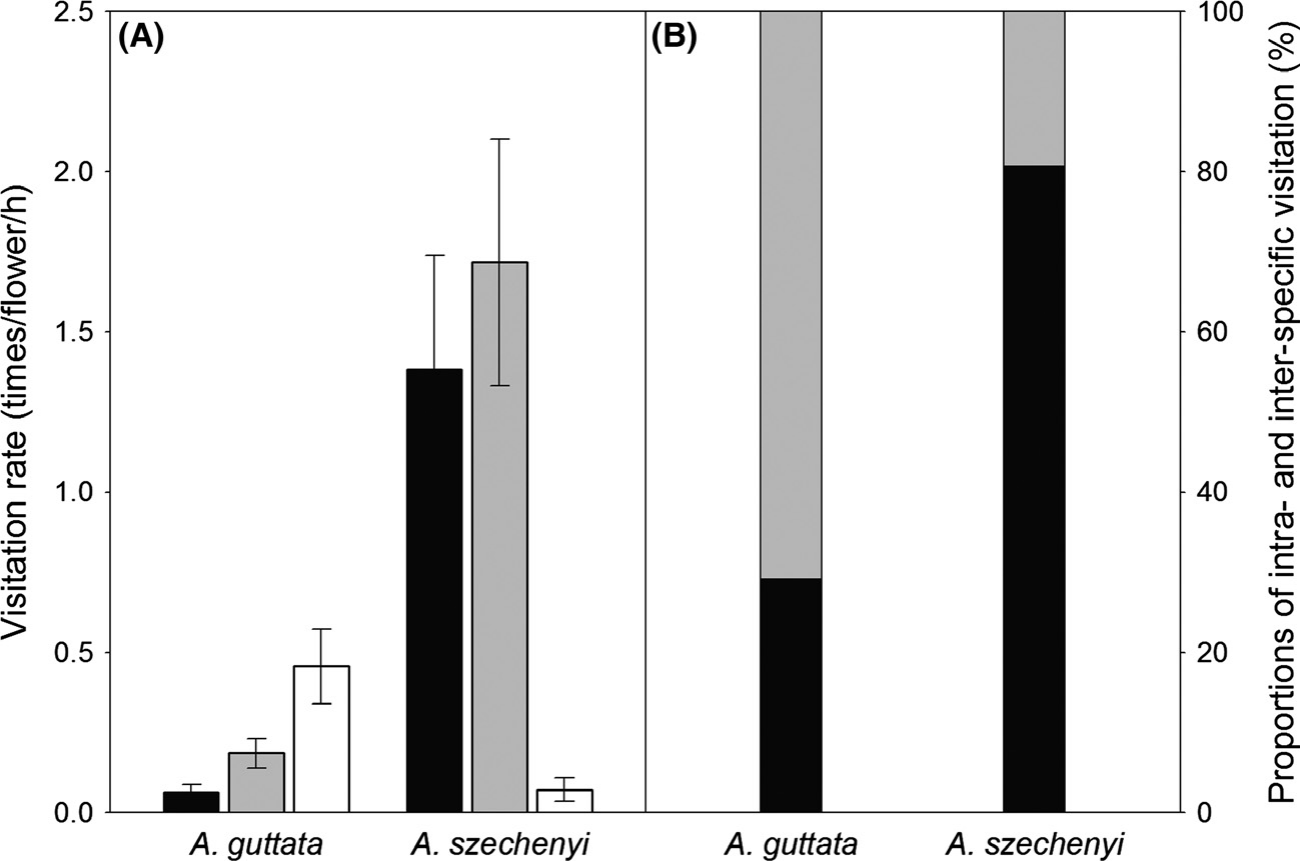
Pollinator visitation rates (A, Mean ± SE) for *A. guttata* and *A. szechenyi*, and proportions of inter‐ and intraspecific visitations (B). A: black, gray, and white bars represent visits by *Amegilla quadrifasciata*,* Amegilla velocissima,* and *Nomioides minutissimus*, respectively. B: black and gray bars represent intra‐ and interspecific visitations.

Three bee species, *Nomioides minutissimus*,* Amegilla quadrifasciata,* and *Amegilla velocissima*, were identified as pollinators shared by the two *Arnebia* species. *Nomioides minutissimus* displayed a preference for visiting *A. guttata*, but *A. quadrifasciata and A. velocissima* exhibited preferences for *A. szechenyi* (Fig. 3A). Generally, the visitation rate was significantly affected by plant species but not pollinator type, and a significant species × pollinator interaction effect was detected, indicating that the presence of both *Arnebia* species changes the pollinators’ visitation patterns (Table 3). In general, the visitation rate was significantly affected by plant species but not by the pollinator type and the significant effect of the species × pollinator interaction indicates that the coexistence of the two *Arnebia* species changed the pollinator's visitations (Table 3).

**Table 3:** Results of two‐way ANOVA of variations in pollinator visitation rates, with the species and pollinator as fixed factors

Source	Sum of squares	df	*F*	*P*
Species	0.39	1	9.92	0.003
Pollinator	0.13	2	1.62	0.207
Species × pollinator interaction	1.06	2	13.59	<0.001

In the reciprocal transplant experiments we found that pollinators that initially visited *A. guttata* exhibited low fidelity, as approximately 70% of their visits were interspecific (Fig. 3B). In contrast, pollinators that initially visited *A. szechenyi* exhibited high fidelity, as only ca. 20% of their visits were interspecific (Fig. 3B).

In the hand‐pollination experiments we found that only pollen grains from flowers of the same species but different morphology germinated on the stigmas, and hence could participate in successful seed production. Pollen grains from either species did not germinate on stigmas of the other species. Thus, no seed was produced in flowers subjected to interspecific hand pollination, indicating complete pollen incompatibility between the *Arnebia* species.

### Selective pressure acting on floral traits

In *A. guttata*, we detected significant positive directional selection pressure on flower diameter mediated via both male (*P* < 0.01) and female (*P* < 0.05) fitness, as estimated by pollen export and seed set, and negative selection pressure on floral tube diameter mediated via male fitness (*P* < 0.01) (Table 4). In *A. szechenyi*, we observed significant positive directional selection pressure on nectar guide size via male fitness (*P* < 0.05), together with negative selection pressure on floral tube length (*P* < 0.05) and positive selection pressure on long‐styled flowers via female fitness (*P* < 0.05) (Table 4). Comparisons of the two species indicate that conflicting selective pressures were acting on their floral tube length, flower diameter and nectar guide size via male fitness, and conflicting selection pressure via female fitness were acting on their flower diameter and floral tube diameter.

**Table 4:** Phenotypic linear selection gradients (± SE) for open‐pollinated flowers of *Arnebia guttata* and *Arnebia szechenyi* mediated via male and female fitness, estimated based on the number of exported pollen grains and seeds set, respectively

Traits	Male		Female	
	*Arnebia guttata*	*Arnebia szechenyi*	*Arnebia guttata*	*Arnebia szechenyi*
Floral tube length	0.10 ± 0.00	−0.21 ± 0.00	−0.15 ± 0.16	−0.40 ± 0.17*
Flower diameter	0.31 ± 0.00**	−0.04 ± 0.00	0.54 ± 0.23*	−0.07 ± 0.21
Floral tube diameter	−0.22 ± 0.00**	0.05 ± 0.00	0.01 ± 0.15	−0.03 ± 0.13
Nectar guide size	−0.14 ± 0.00	0.25 ± 0.00*	0.09 ± 0.20	0.25 ± 0.15
Nectar circle diameter	−0.06 ± 0.00	−0.10 ± 0.00	−0.07 ± 0.27	0.23 ± 0.22
Flower morph	0.00 ± 0.00	0.00 ± 0.00	0.43 ± 0.29	0.55 ± 0.25*

*P < 0.05, **P < 0.01.

## Discussion

The objectives of this study were to explore mechanisms involved in the reproductive isolation of sympatric populations of *Arnebia guttata* and *A. szechenyi*, and the relative importance of pre‐ and postpollination barriers. For this purpose, we examined their phylogenetic relationship; differences in their traits, microhabitats, and pollinator‐mediated selection pressures; and effects of hand pollination.

The phylogenetic analysis distinguished two poorly resolved clades supported by low or moderate low bootstrap values (Fig. 2). One includes *A. guttata*,* A. decumbens,* and *A. linearifolia*, all of which produce yellow flowers (Nasir 1989; Zhu et al. 2005). *Arnebia szechenyi* also produces yellow flowers, but the other species in its clade (*A. fimbriata* and *A. euchroma*) have blue to purple (Zhu et al. 2005). Furthermore, the chromosome numbers (2n) of the species are 16 for *A. linearifolia* (Coppi 2006), 22 for *A. decumbens* (Ghaffari 1996), and 14 for *A. guttata* (Fang and Zhang 1992; Khatoon and Ali 1993), *A. euchroma* (Fang and Zhang 1992), and *A. szechenyi*. Overall, we conclude that *A. guttata* and *A. szechenyi* are closely related species, but further analysis is required to resolve the phylogeny of the genus more thoroughly.

Distributions of the two focal *Arnebia* species display a pattern of geographical substitution, with a sympatric region extending from northwest Gansu to southern Inner Mongolia (Fig. 1A) (Zhu et al. 2005). We hypothesized that this could be causally linked to the increase in precipitation from northwest to southeast China (Piao et al. 2003), if *A. guttata* is less drought tolerant than *A. szechenyi*. This hypothesis was corroborated by findings that *A. guttata* has larger vessel cells than *A. szechenyi*, as large vessel cells are associated with high rates of water and nutrient transport to above‐ground parts of plants, and low drought tolerance (Mengel et al. 2001). Furthermore, *A. guttata* occupied drier microhabitats than *A. szechenyi* at the sympatric sites examined. However, this difference in microhabitats could not contribute strongly to the reproductive isolation of the sympatric populations because the two species were very close to each other at these sites.

Prepollination mechanisms are considered to be more important generally than postpollination mechanisms in the reproductive isolation of sympatric populations, because they should theoretically reduce gamete waste more strongly, and discriminative pollinator visitation is believed to be the main prepollination isolation mechanism (Ramsey et al. 2003; Husband and Sabara 2004; Kay 2006). However, in populations of the two examined *Arnebia* species we observed interspecific visitations, suggesting that shared pollinators do not completely discriminate between their flowers, although the shared pollinators appeared to prefer and visit *A. szechenyi* more loyally than *A. guttata* (Fig. 3). The latter finding is corroborated by the large nectar guides (Table 1) and indications of positive selection pressure on the guide's size in *A. szechenyi* (Table 4). In addition, the evidence of conflicting linear selection pressures acting on all measured floral traits of the two *Arnebia* species, via both male and female fitness (as estimated by pollen export and seed set; Table 4), might reflect disruptive selection that significantly contributes to prepollination reproductive isolation. Thus, despite the substantial frequencies of interspecific visitation by the shared pollinators, pollinator discrimination might contribute to prepollination isolation between the two *Arnebia* species.

Differences in flowering phenology may also contribute strongly to the reproductive isolation of some sympatric species (Salvolainen et al. 2006). We did not quantify the flowering phenology of the two *Arnebia* species, but we observed extensive overlap in their flowering times, indicating that it probably contributes little to prepollination reproductive isolation in this case. Collectively, our results suggest that reproductive isolation of *A. szechenyi* and *A. guttata* is largely mediated by interspecific incompatibility between pollen and stigmas in the sympatric populations. If so, they provide one of the few cases of postpollination prezygotic isolation of a closely related pair of sympatric species, which is believed to be uncommon because of the small genetic distances between closely related plants.

## Conflict of Interest

None declared.

## Supporting information


**Table S1.** Information on the species, populations and sequences included in the phylogenetic analysis.
**Figure S1.** Fluorescence histograms illustrating the nuclear DNA contents of *A. guttata* (A) and *A. szechenyi* (B) obtained by flow cytometric analysis of propidium iodide‐stained nuclei.
**Figure S2**. Vascular phenotypes of the roots of *A. guttata* (A, C) and *A. szechenyi* (B, D). C and D are magnified views of the boxed regions shown in A and B, respectively. X, xylem; Ph, phloem. Bars: 50 *μ*m in (A, B) and 10 *μ*m in (C, D).
