# Supplementary material for: Reproductive isolation is mediated by pollen incompatibility in sympatric populations of two Arnebia species
Source: Ecol Evol. 2015 Nov 26;5(24):5838–46. doi: 10.1002/ece3.1849 (PMC4717334; doi:10.1002/ece3.1849)
Supplement: Supplementary file 1 — Table S1. Information on the species, populations and sequences included in the phylogenetic analysis. Figure S1. Fluorescence histograms illustrating the nuclear DNA contents of A. guttata (A) and A. szechenyi (B) obtained by flow cytometric analysis of propidium iodide‐stained nuclei. Figure S2. Vascular phenotypes of the roots of A. guttata (A, C) and A. szechenyi (B, D). C and D are magnified views of the boxed regions shown in A and B, respectively. X, xylem; Ph, phloem. Bars: 50 μm in (A, B) and 10 μm in (C, D). [file ECE3-5-5838-s001.docx]

Wang et al. Reproductive isolation is mediated by pollen incompatibility in sympatric populations of two *Arnebia* species

Additional files

Table S1. Information on the species, populations and sequences included in the phylogenetic analysis.

| Species | Pop. | N | Location or Accession No. | Haplotype |
| --- | --- | --- | --- | --- |
| *A. szechenyi* | AS-Z | 12 | Zhangye, Gansu | H1 |
|  | AS-Y | 12 | Yabulai, Inner Mongolia | H2, H3, H4, H5, H6 |
| *A. guttata* | AG-Z | 12 | Zhangye, Gansu | H7 |
|  | AG-Y | 12 | Yabulai, Inner Mongolia | H7 H8 |
| *A. fimbriata* | - | 3 | Helan, Ningxia | - |
| *A. euchromae* | - | 1 | EF199860 | - |
| *A. decumbens* | - | 1 | EU919579 | - |
| *A. linearifolia* | - | 1 | EU919580 | - |
| *O. paniculat* | - | 1 | EF199859 | - |


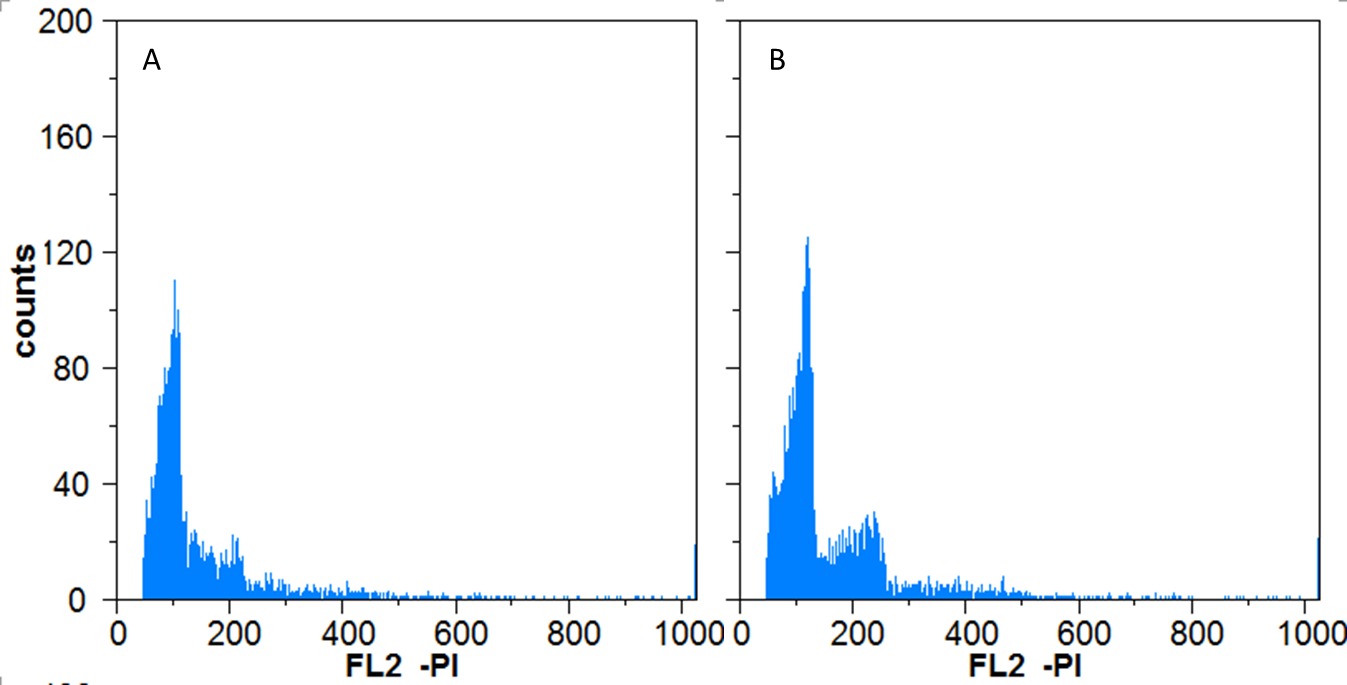


Figure S1. Fluorescence histograms illustrating the nuclear DNA contents of *A. guttata* (A) and *A. szechenyi* (B) obtained by flow cytometric analysis of propidium iodide-stained nuclei.


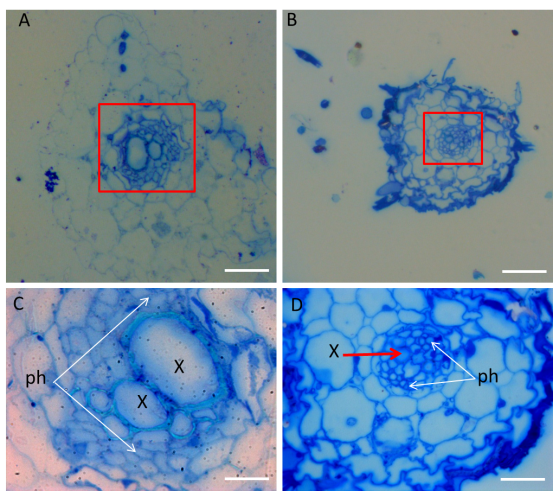


Figure S2. Vascular phenotypes of the roots of *A. guttata* (A, C) and *A. szechenyi* (B, D). C and D are magnified views of the boxed regions shown in A and B, respectively. X, xylem; Ph, phloem. Bars: 50 µm in (A, B) and 10 µm in (C, D).
